# Chirality Effect on Physical and Biological Properties of Peptide-Based Hydrogels

**DOI:** 10.3390/gels12050399

**Published:** 2026-05-05

**Authors:** Lucia De Rosa, Luca Domenico D’Andrea, Alessandra Romanelli

**Affiliations:** 1 Istituto di Biostrutture e Bioimmagini, Consiglio Nazionale delle Ricerche (CNR), Via P. Castellino 111, 80131 Napoli, Italy; lucia.derosa@cnr.it; 2 Istituto di Scienze e Tecnologie Chimiche “G. Natta”, Consiglio Nazionale delle Ricerche (CNR), Via M. Bianco 9, 20131 Milano, Italy; 3 Dipartimento di Scienze Farmaceutiche, Università degli Studi di Milano, Via Venezian 21, 20133 Milano, Italy

**Keywords:** peptide, self-assembling, hydrogel, chirality, supramolecular, D-amino acid

## Abstract

The self-assembly of peptide-based building blocks into ordered structures is widely exploited for the development of novel biomaterials, including hydrogels. In this review, we analyze the effect of chirality on the ability of peptides to form hydrogels. We describe systems composed of peptides of opposite chirality i.e., peptides composed of all L- or D-amino acids and peptides composed of amino acids with alternate chirality, i.e., one L- and D-amino acid or one block containing all L-amino acid followed by one block composed of all D-amino acids. Finally, we illustrate systems composed of mixtures of L- and D-peptides. The structural features of these compounds are discussed. We further compare the mechanical properties of hydrogels formed by homochiral and heterochiral peptides. Finally, we discuss the potential biological applications of these systems, focusing on the differences between hydrogels formed from peptides of opposite chirality or mixed chirality.

## 1. Introduction

Peptides are versatile building blocks for the development of hydrogels; they are biocompatible, easy to produce, and show properties such as structure and biological activity that are strictly dependent on the sequence [1]. Hydrogel formation occurs when peptides self-assemble into supramolecular structures stabilized by intermolecular backbone hydrogen bonding and the side-chain interactions, with the resulting assemblies interacting with water to form a hydrated network. In many cases, these supramolecular structures adopt morphologies such as fibers or tubes. The length of these elongated structures, as well as the degree of interconnection within the fibrillar network, correlates to the mechanical properties of the hydrogels.

The design of hydrogel-forming peptides is a challenging task, and various computational tools have been developed to support it. Recently, the combination of machine learning tools and experimental data has enabled the determination of a score function for the discovery of hydrogelator tetrapeptides [2], and more broadly data driven methodologies have been applied to the de novo design of hydrogels [3]. However, these approaches primarily focus on sequence composition and physicochemical properties, while the role of chirality in modulating the gelation propensity of peptides remains largely unexplored.

Peptide sequences forming hydrogels comprise hydrophobic and aromatic residues that promote self-assembly via van der Waals and π-π interactions, along with polar residues and hydrogen bonding groups that generate amphiphilic structures promoting interactions with water. For example, the combination of a strong self-assembling motif, such as FF or YF, with a lysine residue produces effective gelators: KFF or KYF form hydrogels, unlike PFF [4]. KFF and KYF assemble into fibers; the polar residue allows the interaction of the fiber with water. A further example of hydrogel-forming peptides comprises sequences containing aliphatic residues and terminating with a polar residue, such as Ac-LIVAGD [5]. This peptide assembles into amyloid type fibers, formed through a stepwise process in which the peptide conformation changes from random, to α-helical, and then to a cross β-structure [6]. Gelation also occurs with peptides composed of alternating hydrophobic and hydrophilic residues, such as EAK16 or RADA16, which are stabilized by electrostatic interactions [7].

In this review, we describe selected examples of peptide-based hydrogels: we analyze the effect of chirality on the secondary or three-dimensional structure of peptides, the morphology of supramolecular structures, the mechanical properties of hydrogels and finally, where available, we report chirality-related biological effects.

## 2. Chirality in Peptide-Based Hydrogels

**Figure 1:**
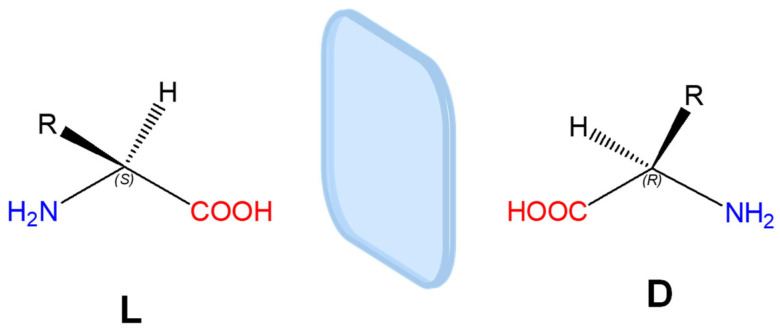
Chemical structure of a generic chiral amino acid (R ≠ H), with the indication of the corresponding L or D configuration.

Peptides are composed, with few exceptions, of chiral monomeric units. In amino acids, the α-carbon bears four substituents, and when all four are different, two stereochemical configurations, denoted as L or D, are possible. L- and D-amino acids are enantiomers, i.e., molecules whose mirror images are not superimposable (Figure 1). Natural, ribosomally synthesized proteins are composed exclusively of L-amino acids; D-amino acids are occasionally found in non-ribosomal natural peptides due to enzymatic processes.

Of course, synthetic chemists can prepare peptides containing both L- and D-amino acids, and in recent years, mirror-image proteins (proteins composed exclusively of D-amino acids) have been synthesized using native chemical ligation and related methodologies [8,9].

Amino acid chirality can be indicated using the L and D notation, which refers to the relative configuration of the stereogenic center with respect to L- and D-glyceraldehyde, or by the Cahn-Ingold-Prelog (R/S) system, although the latter is less commonly used for amino acids. In the R/S system, natural L-amino acids almost always correspond to the S configuration, with the exception of L-cysteine, which is assigned to the R configuration due to the higher priority of the sulfur atom relative to the carboxyl group.

**Figure 2:**
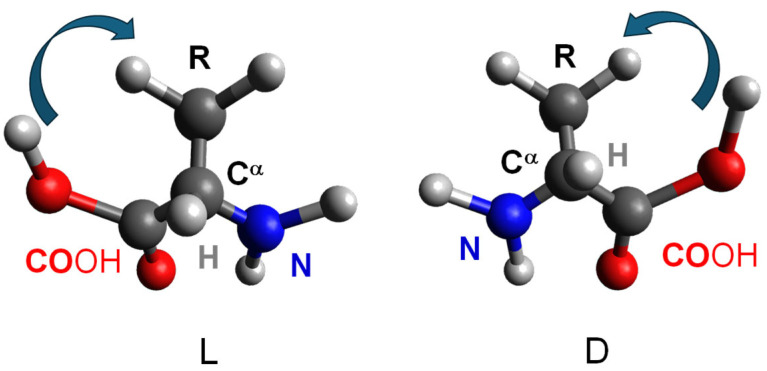
The determination of amino acid chirality using the “corn-crib” rule. The α-hydrogen (shown in grey) is oriented toward the observer. The carbonyl group (or carboxylic as in figure) is represented as CO, the side chain as R, and the α-amino group as N. For an L-amino acid, the sequence CO–R–N is read clockwise, whereas for a D-amino acid, it is read counterclockwise.

A straightforward way to recognize the handedness of an amino acid (or a residue within a biomolecule) is the “corn-crib” rule, originally described by J. Richardson (Figure 2) [10].

For an L-amino acid, when looking along the direction of the H–Cα bond with the hydrogen atom oriented toward the observer and the carbon atom behind, the remaining substituents, the carbonyl (CO), side chain (R), and amino group (N), are arranged such that the sequence CO–R–N reads clockwise.

While most amino acids contain a single stereogenic center at the α-carbon, additional chiral centers may be present within the side chain, as in threonine (Cβ) and isoleucine (Cγ). In these cases, the R/S system is preferred to unambiguously define the configuration of each stereocenter, e.g., (2S,3R)-threonine.

### Effect of Amino Acid Handedness on Peptide Conformation

**Figure 3:**
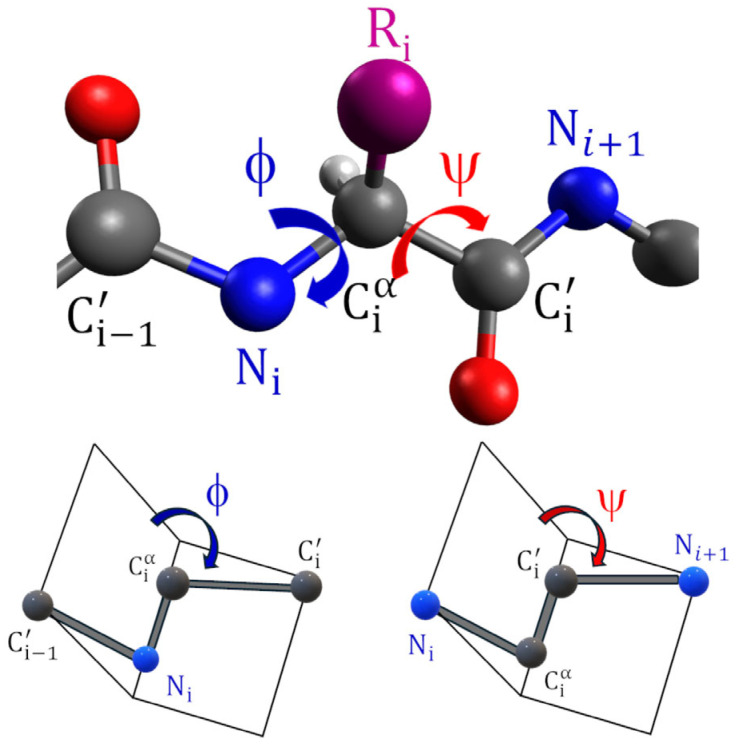
Definition of peptide backbone dihedral angles. The subscript *i* refers to the *i*-th amino acid. Double bonds and hydrogen atoms have been omitted for clarity.

The peptide main-chain conformation is defined by the sequence of backbone dihedral angles, denoted as ϕ (phi) and ψ (psi), which correspond to rotations around the N-Cα and Cα-C′ bonds, respectively (Figure 3).

**Figure 4:**
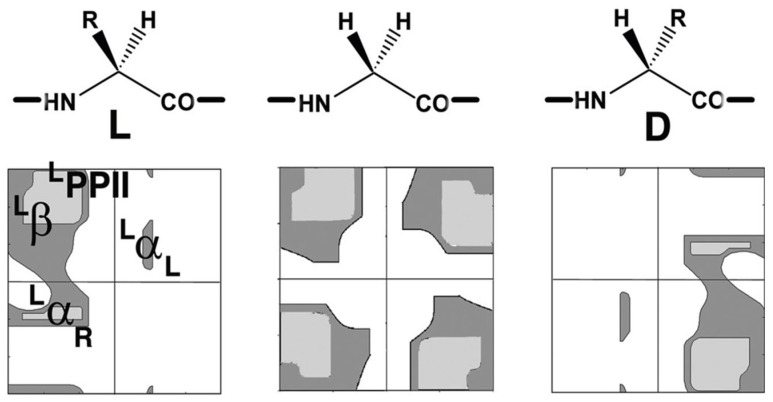
Ramachandran plots of a generic amino acid (excluding proline): L-amino acid (**left**), glycine (**center**), and D-amino acid (**right**). Allowed regions are shown as grey areas and are indicated in the left panel. ^L^α_R_: right-handed helix; ^L^α_L_: left-handed helix; ^L^β: beta-structure; ^L^PPII: polyproline II helix. Adapted with permission from S. Durani, Protein Design with L- and D-α-Amino Acid Structures as the Alphabet, Accounts Chem Res 41 (10) (2008) 1301–1308 [13]. Copyright 2008 American Chemical Society.

Steric constraints limit free rotation around these two bonds, severely restricting the range of accessible ϕ and ψ values [11]. The allowed combinations of ϕ and ψ angles are conventionally represented in a Ramachandran plot, which provides a visual map of sterically favorable conformations (Figure 4) [12].

Comparison of Ramachandran plots of L- and D-amino acids clearly shows that the two enantiomers populate distinct regions of conformational space that are related by mirror symmetry. Glycine represents a special case: being achiral, it exhibits enhanced conformational flexibility and can access regions characteristic of both L- and D-amino acids.

Amino acid chirality has a profound impact on the spatial organization of peptide secondary structures. Helices composed of L-amino acids almost exclusively adopt a right-handed screw sense, as left-handed helices are energetically disfavored due to steric clashes between side chains and backbone carbonyl groups. Conversely, helices composed of D-amino acids preferentially assume left-handed conformations. Similar principles apply to higher-order assemblies such as coiled coils. In canonical coiled coils composed of L-amino acids, right-handed α-helices wrap around each other to form a left-handed supercoil [14]. This inversion of handedness mainly arises from packing effects. Mirror-image coiled coils assembled from D-amino acids display the opposite architecture, consisting of left-handed α-helices forming right-handed supercoils.

In β-sheet structures, amino acid chirality affects the intrinsic twist of the β-strands, with L-amino acids favoring a right-handed twist, which in turn produces a left-handed spiral in the β-sheet [15,16,17]. β-turns, which connect two antiparallel β-strands, also exist in mirror-related conformations (e.g., Type I vs. Type I′), in which the peptide backbone adopts strictly mirror-image geometries. D-amino acids preferentially promote the formation of mirror-beta turns [18]. Homochiral β-strands, composed exclusively of L- or D-amino acids, align into geometrically compatible hydrogen-bonding arrays, forming planar β-sheet architectures. In contrast, when β-strands of opposite chirality are juxtaposed, the stereochemical mismatch prevents the formation of a flat sheet. These assemblies instead adopt a periodically undulated topology known as a rippled β-sheet. In such structures, alternating peptide strands with L- and D-configuration give rise to the characteristic rippled surface, as originally predicted by Pauling and Corey [19]. Co-assembly into rippled β-sheets seems to be enthalpically driven in amphipathic peptides with alternate hydrophobic and hydrophilic amino acids [20]. In other cases, self-sorting instead of co-assembly occurs. As an example, mixtures of enantiomeric amyloid peptides form mixtures of distinct L and D enantiomeric fibrils [21]. A recent study on self-assembling peptide amphiphiles shows that self-sorting occurs when peptides forming right-handed nanostructures are mixed to peptides forming left-handed structures, likely due to an enthalpic penalty required for changing the twist of the β-sheet [22]. On the contrary, peptides that form either left- and right-handed nanostructures can co-assemble with either peptide.

Heterochiral helical peptides, composed of segments with opposite chirality, retain the helical handedness dictated by the local amino acid chirality. Consequently, the helix undergoes a reversal of screw sense between segments, generating a structural discontinuity at the hinge site [23,24].

Peptide hydrogel formation is a hierarchical and multiscale process that spans several levels of molecular organization. It begins at the molecular level, with peptide folding into defined secondary structural elements such as β-sheets, β-hairpins, or α-helices, which can further organize into tertiary-like arrangements stabilized by intramolecular hydrogen bonding, hydrophobic interactions, and side-chain packing [25,26,27,28]. These folded peptides subsequently undergo self-assembly into ordered supramolecular nanostructures, including nanotubes, nanowires, nanobelts, ribbons, and fibrillar architectures, depending on the peptide sequence, concentration, and environmental conditions. When these supramolecular peptide-based nanostructures exhibit a helical twist, their handedness can be classified according to the IUPAC helicity convention: structures adopting a clockwise progression of the helical contour along the principal axis are designated as P (plus, right-handed) helices, whereas those displaying a counterclockwise progression are designated as M (minus, left-handed) helices. At the next hierarchical level, these supramolecular building blocks interact and entangle to form a three-dimensional network that immobilizes large amounts of water, thereby giving rise to a macroscopic hydrogel [29].

Chirality plays a central role throughout the self-assembly process, particularly during the formation of supramolecular nanostructures. The intrinsic chirality of amino acids and the resulting handedness of peptide secondary structures are often propagated, leading to chiral supramolecular organization. For example, peptide nanofibers, commonly observed as the primary structural elements in peptide-based hydrogels, frequently adopt twisted or helical morphologies. Individual protofilaments or ribbons can laterally associate and wrap around each other, forming higher-order fibers with a defined screw sense, either right-handed or left-handed. This supramolecular chirality is typically dictated by the stereochemistry of the peptide building blocks, the preferred twist of β-sheets or helices, and the balance between hydrogen bonding, hydrophobic interactions, and electrostatic forces [30,31]. Importantly, chiral amplification can occur during assembly, whereby subtle stereochemical preferences at the molecular level are translated into pronounced macroscopic chirality at the nanofiber and network scales. This hierarchical transfer of chirality is a defining feature of peptide hydrogel systems and represents a key organizing principle governing their structural and mechanical properties.

## 3. Effect of Chirality on Physical and Mechanical Properties of Hydrogels

The chirality of amino acids plays a key role in determining not only the secondary structures of peptides but also their solubility in aqueous media and their ability to form supramolecular structures able to interact with water to produce hydrogels. The physical properties of hydrogels, such as stiffness and porosity, are closely related to the morphology of the supramolecular assembly. Homochiral peptides of opposite chirality form secondary structures as mirror images, and the physical properties of the resulting hydrogels are generally comparable. In contrast, heterochiral peptides assemble into different structures compared to homochiral peptides, yielding hydrogels with diverse/divergent mechanical and physical properties. The impact of heterochirality on solubility and aggregation can be rationalized in terms of its effects on backbone geometry, side-chain orientation, and intermolecular interactions. The incorporation of D-amino acids into predominantly L-peptide sequences alters the φ/ψ dihedral angle preferences and influences the regular spatial arrangement of side chains, thereby modifying both folding and intermolecular packing. The effect of heterochirality on gelation is particularly evident in short peptides which lack sufficient length to form stable intramolecular structures, where these perturbations often favor intermolecular association. In particular, heterochirality can increase the exposure to the solvent of hydrophobic residues and reduce favorable peptide–solvent interactions, effectively lowering solubility and promoting aggregation. The reduced conformational bias of short heterochiral sequences may also facilitate access to aggregation-prone conformations, further enhancing self-association. In longer sequences, however, the formation of ordered β-sheet structures requires the precise alignment of backbone hydrogen bonds and side-chain packing. The introduction of opposite chirality residues disrupts these geometric constraints, leading to mismatches in strand registry and suboptimal hydrogen bonding. As a result, the system experiences packing frustration, where competing structural preferences cannot be simultaneously satisfied, ultimately giving rise to increased structural disorder, heterogeneous assemblies, or alternative supramolecular organizations.

Here, we describe hydrogels formed by homochiral and heterochiral peptides, classified according to their length, with the aim of highlighting the structural features that determine hydrogel formation and properties. Moreover, we report selected examples in which chiral structures are obtained using non-standard amino acids or by conjugating organic molecules or polymers to amino acids and peptides.

### 3.1. Analysis of Short Peptide-Forming Hydrogels

#### 3.1.1. Dipeptide-Forming Hydrogels

Studies on dipeptides mainly focus on hydrophobic peptides, typically containing at least one F residue. In these cases, the chirality of the amino acids affects the peptide hydrophobicity, with heterochiral peptides generally being less soluble, more hydrophobic than homochiral peptides and therefore more prone to forming hydrogels. This phenomenon can be explained by considering that a change in amino acid chirality induces a conformational modification in the dipeptide backbone as the preferred main-chain dihedral angles have opposite signs for L- and D-amino acids, as discussed in the previous section. Consequently, the relative orientation of the hydrophobic sidechains and the direction of the peptide bond dipoles are altered, affecting the exposure of hydrophobic surfaces and the net dipole moment, respectively. The gelation process in dipeptides is associated with the formation of nanotubes that can incorporate water. The dimensions of these nanotubes depend primarily on the peptide sequence and, in some cases, on chirality. Here, we discuss the properties of hydrogels formed by uncapped and capped peptides.

The FF sequence, known as the key assembly unit of the β-amyloid polypeptide, is one of the most widely investigated low molecular weight gelators [32,33,34]. The homochiral peptide forms parallel β-structures, with peptides held together by head-to-tail ionic interactions, producing rigid microtubes with heterogeneous diameters [35]. These assemblies yield a metastable hydrogel, which undergoes syneresis overtime and is not thermoreversible [36]. Hydrogel formed by the heterochiral peptide fF has been investigated by Kralj and coworkers in 2020 [37]. The dipeptide fF arranges into fibrils of homogeneous size with diameters of approximately 4 nm, forming a transparent hydrogel. The resistance to stress of the heterochiral peptide is superior to that of the homochiral peptide (Table 1). X-ray diffraction (XRD) analysis of the nanotubes formed by homo- and heterochiral peptides has revealed similar supramolecular packing, with stacked peptides, similar patterns of hydrogen bonds, and salt bridges. The chirality of the N-terminal residue dictates the helical handedness along the tube. As a result, the homochiral FF assembles into a left-handed helical supramolecular structure, whereas the heterochiral fF shows the opposite handedness, supporting the observation that beta-structures composed of L-amino acids self-assemble with a left screw sense. The main difference between the assembled homo- and heterochiral peptides lies in the orientation of the aromatic rings, resulting from the change in chirality and the different backbone dihedral angles. In homochiral FF peptide, the two phenylalanine side-chain rings interact in an edge-to-face orientation generating a rougher surface with a wider hydrophobic interface area which promotes the bundling of tubes. In contrast, in heterochiral fF, the aromatic rings adopt face-to-face orientation, producing a smoother surface that does not favor bundling, increasing, instead, the intramolecular hydrophobic area between phenylalanine side chains. The solvent accessible surface area is larger for the heterochiral peptide than for the homochiral one, resulting in a homogeneous and transparent hydrogel. Hydrogel stability is related to the order of fibers. In cell culture medium, the stability of the fF hydrogel was higher compared to that of FF. Moreover, the biological properties of the homo- and heterochiral gels are distinct.

Other examples of hydrophobic uncapped peptides are dipeptides composed of leucine and phenylalanine of the same or opposite chirality [38]. All peptides, with the exception of FL, form hydrogels. In transmission electron microscopy (TEM), FL appears as an amorphous aggregate, while crystal structure analysis reveals the formation of nanotubes and the absence of interdigitated aromatic side chains. The heterochiral fL forms a metastable gel, appearing in TEM as a mixture of crystals and short fibers. The X-ray structure reveals the formation of alternating hydrophilic and hydrophobic layers, in which the aromatic side chains of phenylalanine from adjacent layers do not interact. The absence of interlayer Phe-Phe zipping, which would otherwise generate a continuous hydrophobic interface that excludes water, appears to be a key factor affecting the gel formation and stability (FL does not form hydrogel, fL forms a metastable gel). The inversion of the chirality of Phe induces a conformational frustration, resulting in a structure in which hydrophobic interactions are localized within individual layers and do not propagate across them. However, this lack of interlayer hydrophobic cohesion is partially compensated by the incorporation of water molecules, which act as essential bridging elements through hydrogen-bonding networks linking N-termini, amide carbonyl groups, and C-termini, thereby enabling hydrogel formation, albeit in a metastable form [38]. By inverting the position of Leu and Phe in the dipeptide sequence (LF peptide), both LF homo- and heterochiral peptides form hydrogels, with gelation occurring faster for the heterochiral peptide than for the homochiral one. X-ray analysis of LF dipeptide reveals the presence of nanotubes, interdigitated with each other into zippers. LF and lF in TEM appear as heterogeneous fibers. As observed for FF and fF, the formation of ordered supramolecular structures is a key factor driving hydrogel formation.

Cyclization of LF, lF, Lf into diketopiperazines produces more hydrophobic compounds and eliminates the possibility of head-to-tail electrostatic interactions compared to the corresponding linear dipeptides [39]. While cycloLF forms hydrogel in PBS, the heterochiral peptides precipitate under the same conditions. This difference in behavior may be attributed to the ability of the homochiral dipeptide to adopt an amphipathic structure, as suggested by structural studies, which promotes ordered self-assembly. In contrast, heterochiral peptides do not exhibit a net segregation between hydrophobic and the hydrophilic regions, likely due to stereochemically induced packing constraints, and therefore fail to form hydrogels. Studies on the cyclic FF and fF have revealed a similar behavior [40].

Studies on hydrogel formed by dipeptides containing isoleucine and phenylalanine, with the same or opposite chirality, have been described [41]. The isomers IF, iF, FI and fI have been investigated as representative of their mirror images, as the supramolecular arrangement of enantiomers is identical. Heterochiral compounds are more hydrophobic than homochiral ones, suggesting a different spatial organization of the side chains and backbone. The peptides IF, iF and FI do not form hydrogels in PBS, whereas the heterochiral fI produces microfibers and an opaque gel. Its elastic modulus is higher than that of the hydrogel formed by its peptide isomer fL (Table 1). TEM micrographs reveal that only fI peptide produces long fibers, whereas the other dipeptides form short fibers (<2 microns) with limited interconnectivity. X-ray structure reveals that only the gelator dipeptide fI forms nanotubes similar to those of FF, held together by head-to-tail interactions, among six peptides, resulting in an amphipathic water channel. On the contrary, the non-gelling peptides are organized into amphiphilic layers.

Homochiral dipeptides composed of valine and phenylalanine show a behavior similar to the dipeptide composed of isoleucine and phenylalanine. The VF and FV peptides are unable to form hydrogels due to their low hydrophobicity [40,42]. The heterochiral peptide fV is more hydrophobic than their homochiral counterparts and prone to fibrillation [43] X-ray data reveal that fV peptides assemble into nanotubes: four peptides are held by head-to-tail interactions, resulting in a channel filled with water. Consistent with these observations, TEM analyses reveal the presence of fibers for the gelling fV peptide. Analysis of the stability of the fV hydrogel in cell culture media reveals that it is limited, similarly to what is observed for fF hydrogels. In contrast, unlike the other heterochiral peptides, vF peptides do not yield hydrogels. TEM analysis reveals that vF forms amorphous aggregates, unlike fV. XRD data indicate that in the gelling peptides, water molecules interact with extended stack; in the non-gelling peptides water molecules form H-bonds that interconnect different peptides, preventing the formation of a stable hydrogel. These results suggest that the heterochirality in dipeptides is not a sufficient condition to grant the formation of hydrogels. The steric hindrance and the orientation of the side chain determine the packing of the peptides and the formation of ordered or unordered structures.

A widely explored strategy to obtain new gelators consists in the functionalization of a peptide, for example through N-terminal conjugation with an aromatic moiety [44,45,46]. Few examples of heterochiral capped peptides able to form hydrogels have been reported in the literature. Recent studies describe the gelation of fluorenylmethoxycarbonyl (Fmoc)-protected FF, ff, fF, and Ff. The secondary structure of the peptides in the fibers was investigated by CD: all peptides adopt a β-sheet conformation, with slight differences in twist degree, likely due to the different aggregation pathways. Hydrogel formation was monitored by time-lapse optical microscopy imaging: gelation occurs when spheres that initially form turn into fibrils. This process occurs rapidly for homochiral peptides and is slower for heterochiral peptides [47]. The kinetic of fibrillization is different for the two hetero-enantiomers, being slower for Fmoc-Ff. In addition, the morphology of Fmoc-fF appears feather-like, in contrast to the more compact morphology of Fmoc-Ff. The homochiral Fmoc-FF and Fmoc-ff peptides show very high storage moduli (Table 1). The heterochiral peptides show different stiffness: Fmoc-Ff has a lower G’ than Fmoc-fF (Table 1). As observed for fV and vF, also in this case the packing of the molecules determines a different supramolecular structure, which results in different mechanical properties.

In another example, homochiral FF and heterochiral Ff conjugated to a Peptide Nucleic Acid dimer have been investigated [48]. The conjugates form hydrogels when mixed with the peptides FF and Ff. These hydrogels, named FFmix and Ffmix respectively, display similar mechanical properties, as revealed by rheological analyses (Table 1). Scanning Electron Microscopy (SEM) measurements reveal the presence of highly interconnected fibers in all cases. Interestingly, Small-Angle X-ray Scattering (SAXS) measurements indicate a difference in porosity between the homochiral and heterochiral hydrogels, likely due to variations in the degree of interconnections between fibers.

**Figure 5:**
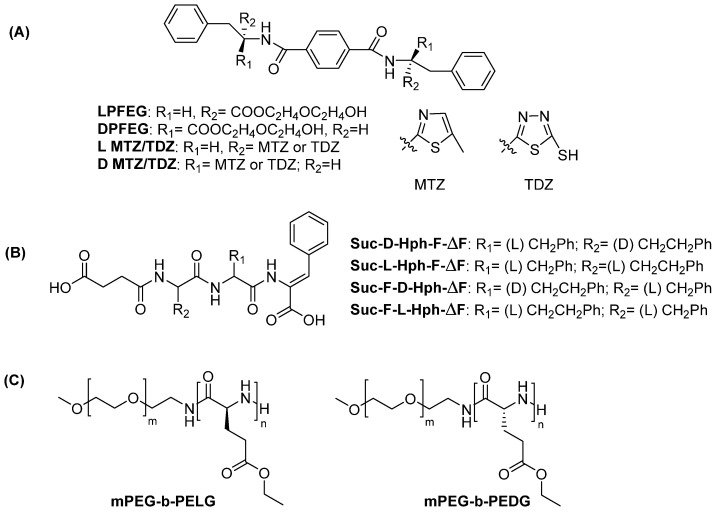
Chemical structures and names of selected modified peptides. (**A**) 1,4 benzendicarbonylcarboxyamide scaffold-based peptides; (**B**) capped tripeptides. Suc = succinyl; Hph = homophenylalanyl; ΔF = dehydrophenylalanyl; (L) and (D) refer to the residue configuration.; (**C**) PEG-based block copolymers.

Following a different strategy, hydrogelators can be obtained by conjugating phenylalanine to hydrophobic, planar molecules. The 1,4 benzendicarbonylcarboxyamide scaffold, conjugated to D- or L-phenylalanine-diglycol derivatives, has been largely investigated for hydrogel formation [49,50,51]. When the amino acid is in the L configuration, the derivative named LPFEG yields left-handed nanofibers (M-type). On the contrary, when the amino acid is in the D configuration, the derivative named DPFEG, forms right-handed nanofibers (P-type). The chemical structures of LPFEG and DPFEG are depicted in Figure 5A. These compounds form porous hydrogels. The storage and loss modulus of the hydrogels with different chirality are similar (Table 1). Importantly, these hydrogels are stable in cell culture media, allowing an extensive exploration of their applications as biomaterials (see Section 4). The chirality of the supramolecular structures can be tuned by modifying the distance between the amino acid and the benzendicarboxyamide scaffold: when the distance between the aromatic ring and the carbonyl is one (or an odd number) of methylene units, left-handed helices are formed; when the number of methylene units is even, right-handed helices are formed [52]. These differences in supramolecular chirality result in hydrogels that perform differently in terms of supporting cell adhesion and proliferation.

DPFEG hydrogels were turned into stimuli responsive materials by mixing the peptide units with-graphene oxide (GO). The resulting DPFEG-GO hydrogels were exploited as delivery systems for cancer drugs [53]. GO is a stimuli-responsive material commonly used in photothermal therapy. Upon irradiation at NIR frequencies, the DPFEG right-handed helix (P-type) turns into a left-handed helix. The R-isomer of the drug oxaliplatin is specifically loaded by the hydrogel and is released after irradiation. Rheological analysis of the DPFEG-GO composite reveals that the addition of GO results in an increase in both storage and loss modulus as compared to DPFEG hydrogels.

Replacement of the glycol (EG) unit in DPFEG or LPFEG with 2-amino-5-methylthiazole (MTZ) or 5-amino-1,3,4-thiadiazole-2-thiol (TDZ) yields the corresponding L- or D-MTZ and L- or D-TDZ (Figure 5B). L-MTZ and L-TDZ form left-handed fibers, whereas D-MTZ and D-TDZ form right-handed fibers [54]. The enantiomeric fibers show the same diameter. Rheological studies reveal that both hydrogels have similar resistance to external forces. G′ values were higher for the D enantiomers, suggesting better viscoelastic properties (Table 1). The D-hydrogel shows higher antibacterial activity as compared to the L counterpart.

**Table 1:** Sequences and properties of hydrogels formed by dipeptides.

Peptide Sequence or Name	Secondary Structure	Minimum Gelation Concentration, Solvent	G′ (Pa)	Morphology	Refs.
FF	β-structures	20 mM, PB pH 7.3	22.0 × 10^3^	microtubes	[36,37]
fF	β-structures	20 mM PB pH 7.3	22.9 × 10^3^	fibers	
LF	β-structures	40 mM, PBS	* 10^4^	fiber	[38]
lF	β-structures	40 mM, PBS	* 10^5^	fibers	
fL	β-structures	20 mM, PBS	* 10^3^	crystals&short fibers	
fI	β-structures	20 mM, PBS	7 × 10^4^	microfibers	[41]
fV	β-structures	40 mM, 0.1 M PBS	1.23 × 10^5^	microfibers	[43]
Fmoc-FF	β-sheets	2 mM, 10% DMSO in water	12.8 × 10^3^	nanofibers	[47]
Fmoc-ff	β-sheets	2 mM, 10% DMSO in water	30.4 × 10^3^	nanofibers	
Fmoc-fF	β-sheets	0.4 mM, 10% DMSO in water	23.2 × 10^3^	nanofibers	
Fmoc-Ff	β-sheets	0.4 mM, 10% DMSO in water	766	nanofibers	
FFmix	n.d.	30 mM peptide + 2.5 mM PNA-peptide, 0.1 M PB pH 7.4	25 × 10^4^	fibers	[48]
fFmix	n.d.	30 mM peptide + 2.5 mM PNA-peptide, 0.1 M PB pH 7.4	13.6 × 10^4^	fibers	
LPFEG	n.d.	0.8 mM, 3% DMSO in DMEM	7.4 × 10^3^	helical nanofibers	[49,50,52]
DPFEG	n.d.	0.8 mM, 3% DMSO in DMEM	7.4 × 10^3^	helical nanofibers	
L-MTZ	n.d.	5 mM, DMSO 10% in water	* 10^3^	helical nanofibers	[54]
D-MTZ	n.d.	5 mM, DMSO 10% in water	* 10^3^	helical nanofibers	
L-DTZ	n.d.	4 mM, DMSO 10% in water	* 10^4^	helical nanofibers	
D-TDZ	n.d.	4 mM, DMSO 10% in water	* 10^5^	helical nanofibers	

N.d.: not determined; PB: phosphate buffer; PBS: phosphate-buffered saline; *: estimated from frequency or time or stress sweep graphs reported in the papers.

#### 3.1.2. Tripeptide-Forming Hydrogels

The formation of hydrogels using uncapped tripeptide has been demonstrated by Marchesan and coworkers, using sequences containing combinations of FF with V or L [55,56,57,58]. The homochiral peptides VFF and FFV exist as a mixture of secondary structures or in a disordered conformation and do not form hydrogels in physiological conditions. Changing the chirality of the residue at the N-terminus results in peptides being able to form hydrogels at pH 7.4. The morphology observed at cryo-TEM of the two peptides is different: vFF assembles into long nanotapes, whereas fFV produces twisted nanofibers (Table 2). CD and FT-IR data suggest the formation of antiparallel beta-sheets and the stabilization of the structure by head-to-tail interactions between carboxyl and amino termini. Insights into the effect of chirality inversion on gelification are described in studies on tripeptides LFF and lFF [57]. Only the heterochiral peptide yields a hydrogel; analysis of freshly prepared lFF samples by atomic force microscopy (AFM) reveals the formation of fibers originating from globules. After 24 h, the density of the fibrillar network increases, leading to an increase in the elastic modulus of the hydrogel. XRD and MD analyses on the heterochiral peptide suggest that the phenylalanine zipper contributes to stabilizing the supramolecular structure, resulting in interdigitated beta sheets. On the contrary, the non-gelling homochiral peptide forms only globular structures, as shown by AFM and TEM experiments. In summary, the inversion of the configuration at the N-terminal residue relieves steric clashes between side chains at the strand termini, favoring a more extended β-strand conformation. This structural arrangement facilitates π–π stacking between the aromatic rings of the central phenylalanine, the formation of ionic interactions between peptide termini, and the establishment of extended hydrogen-bonding between amide groups. In addition, the side chains of terminal phenylalanine residues are more exposed and can interdigitate in a knob-into-hole fashion, giving rise to steric zipper-like packing which is critical for stabilizing the self-assembling architecture. This structural motif can further assemble, hierarchically, into a hydrogel.

Further information is reported on FFV in a study aimed at investigating the effect of chirality on the hydrogelation of tripeptides [58]. Eight peptides (four enantiomeric pairs) have been prepared, containing all combinations of D- and L- amino acids. The configuration of the central amino acid drives the chirality of the supramolecular assembly, as assessed by CD spectroscopy. Only peptides with the N-terminal amino acid of different chirality compared to the others (fFV and Ffv) form ordered fibers and gelate. Ffv gelates faster and has a higher elastic modulus than fFV (Table 2). It was hypothesized that these mixed chirality hydrogels differ in fiber density due to different interactions with counterions, which may affect salt bridges formation and, thus, the self-assembling process. No differences in cell viability have been observed.

However, a single inversion of amino acid configuration in tripeptide does not always yield peptides able to form strong hydrogels. For example, in tripeptide VFF, chiral inversion at position #2 (VfF and vFf) results in non-self-supporting hydrogel [59]. Structural characterization revealed that VfF and vFf are inherently more disordered and form assemblies with lower supramolecular order, which are unable to efficiently exclude water molecules. This results in hydrogels with reduced mechanical strength and thermal stability.

Studies on tripeptides with amino acids with alternate chirality of sequence FxF, where x is a D aliphatic amino acid such as Ile, Leu, Val, Nva (norvaline), or Nle (norleucine), reveal that alternating chirality determines the formation of an amphiphilic structure, in which the aliphatic side chain of the D-amino acid is packed between the aromatic rings of Phe, creating a hydrophobic region that excludes water [60]. The kinked backbone defines the hydrophilic region with the charged termini displayed on opposite faces of the backbone. Salt bridges between the N- and C-terminal ends determine the formation of water channels. The phenyl rings zip the water channels. On the contrary, homochiral peptides adopt an extended structure, and no separation between the hydrophobic and hydrophilic faces occurs. These structural differences arise from the inversion of chirality of the central residue, which, as revealed by MD simulations, adopts backbone dihedral angles characteristic of the β-region of a D-amino acid. This inversion promotes a reorientation of the central side chain, positioning it on the same side of the backbone as the flanking Phe residues (reminiscent of a β-turn) thereby generating an amphipathic arrangement that promotes the spatial segregation of hydrophobic and hydrophilic domains and supports the formation of structured aqueous channels. This effect is clearly evident in the X-ray structure of F-DNva-F peptide. In contrast, homochiral tripeptides do not form channel-like structures but instead pack into layered assemblies [60]. Heterochiral peptides form hydrogels. They form fibers of different diameters, depending on the side chain of the central residue x. When x is D-Ile, D-Leu or D-Nle, the hydrogel forms with rapid kinetic and presents a higher elastic modulus as compared to gels in which x is D-Val or D-Nva. When x possesses a linear side chain (D-Nva or D-Nle), thick and rigid fibers form, which negatively affect the resistance of the hydrogel to applied stress. When x possesses a branched side chain (D-Val or D-Ile), thin and flexible interconnected fibers are formed. This results in hydrogels with higher resistance to stress than their linear counterparts (Table 2).

As observed for uncapped dipeptides, in tripeptides the combination of heterochiral amino acids appears to result in peptides with a higher propensity to gelate compared to homochiral ones.

In a recent paper the assembly of enantiomeric heterochiral tripeptides (Hff and hFF) was investigated [61]. Each tripeptide forms amphipathic β-sheets upon assembly; TEM and AFM reveal the presence of fibers. The elastic modulus for the enantiomeric hydrogels was similar (Table 2). Interestingly, when the racemic mixture was evaluated, an increase in the elastic modulus was found. This result is consistent with the co-assembly of the peptides generating rippled beta-sheets.

**Table 2:** Sequences and properties of hydrogels formed by tripeptides.

Peptide Sequence or Name	Secondary Structure	Minimum Gelation Concentration, Solvent	G’ (Pa)	Morphology	Refs.
vFF	β-sheets	** 16.2 mM, 0.1 M PB pH 7.4	* 10^4^	nanotapes	[56]
fFV	β-sheets	** 16.2 mM, 0.1 M PB pH 7.4	* 10^4^	twisted nanofibers	[58]
Ffv	β-sheets	** 17.0 mM, 0.1 M PB pH 7.4	* 10^4^	fibers	
lFF	β-sheets	** 15.7 mM, 0.1 M PB pH 7.4	* 10^4^	fibers	[57]
FiF	β-sheets	10 mM, 0.1 M PB pH 7.3	* 10^3^	fibers	[60]
FlF	β-sheets	10 mM, 0.1 M PB pH 7.3	* 10^3^	fibers	
FnleF	β-sheets	10 mM, 0.1 M PB pH 7.3	* 10^3^	fibers	
FvF	β-sheets	10 mM, 0.1 M PB pH 7.3	* 10^3^	fibers	
FnvaF	β-sheets	10 mM, 0.1 M PB, pH 7.3	* 10^3^	fibers	
hFF	β-sheets	50 mM, PB pH 7.4	34.3 × 10^3^	fibers	[61]
Hff	β-sheets	50 mM, PB pH 7.4	34.9 × 10^3^	fibers	
hFF/Hff	rippled β-sheets	50 mM, PB pH 7.4	5.92 × 10^4^	fibers	
Succ-D-Hph-L-Phe-ΔF	random coils	3.4 mM, D-glucono-δ-lactone	3.35 × 10^3^	fibers	[62]
Succ-L-Hph-L-Phe-ΔF	α-helices and random coils	3.4 mM, D-glucono-δ-lactone	1.75 × 10^4^	fibers with higher twisting, coiling and cross-linking	
Succ-L-Phe-D-Hph-ΔF	β-sheets	3.4 mM, D-glucono-δ-lactone	1.47 × 10^3^	fibers	

N.d.: not determined; PB: phosphate buffer; *: estimated from frequency or time, or stress sweep graphs reported in the reference papers. **: estimated using the calculated molecular weights of the compounds and data reported in the reference papers.

The effect of capping on homochiral vs. heterochiral tripeptides has been demonstrated by Carvalho et al. [62]. Succinylated tripeptides of sequence homophenylalanine (Hph)-phenylalanine-dehydrophenylalanine (ΔF), in which Hph and F have either different chirality (respectively D,L) or identical chirality (L, L), have been investigated, along with peptides obtained from different combinations of these amino acids (Figure 5C). Homo- and heterochiral peptides show a similar propensity to form hydrogels (critical aggregation concentration values are comparable); fibers observed by TEM appear long and thin and are mixed with vesicle-like structures for heterochiral peptides, while thick, ribbon-like structures are observed for homochiral peptides. The homochiral peptide produces a hydrogel stronger than the heterochiral peptide in terms of resistance to strain, but it is less elastic. The presence of Hph and f results in a structure which is different from that observed in peptides containing F, where aromatic rings typically stack. According to molecular dynamic (MD) simulations, interactions between the phenylalanine rings occur in a T-shaped geometry and this may determine the different fiber morphology and the different mechanical properties of the hydrogels.

### 3.2. Analysis of Long Peptide-Forming Hydrogels

The effect of chirality on the gelation properties of long peptides has been explored in different ways. Several examples of hydrogels formed by homochiral peptides of opposite chirality have been reported. In these cases, as also observed for short peptides, the physical and mechanical properties of the hydrogel are comparable [63,64,65,66]. Studies on Ac-FFFK and Ac-fffk tetrapeptides have shown that both peptides, in sucrose solution, produce porous hydrogels with similar mechanical properties (Table 3), exhibiting shear-thinning and self-healing properties [63]. These properties make the hydrogel suitable for injection applications. The peptides are arranged into beta sheets, which in turn form nanofibers. TEM images show the formation of cylindrical nanofibers. Biological properties of the two hydrogels are affected by chirality.

Homochiral peptides of sequence Ac-FFFKTTKS and Ac-fffkttks form hydrogel in a sucrose solution [64]. Peptides composed of L-amino acids assemble into left-handed helical nanofibers, while peptides composed of D-amino acids form right-handed nanofibers. The diameter of both fibers are comparable (13 and 15 nm). The hydrogels exhibit similar mechanical properties (Table 3) and form pores with an average pore size of 53 and 67 μm, which are conducive to cell migration, growth, and differentiation, while showing different biological properties.

The use of a mixture of identical peptides of opposite chirality represents another strategy to modulate the mechanical properties of biomaterials by tuning molecular interactions [67]. This approach, known as stereocomplexation, relies on interactions between complementary stereoregular molecules. The effect of stereocomplexation is sequence specific. When the β-hairpin peptide MAX1 (sequence: (VK)_4_VpPT(KV)_4_)-NH_2_ and the Ac-(FKFE)_2_-NH_2_ peptide, composed of alternating hydrophobic and hydrophilic residues, are blended with their enantiomers, the peptides organize into rippled beta-sheets, while the homochiral peptides form pleated beta-sheets [20,68,69,70]. It has been suggested that the co-assembly of enantiomeric peptides into rippled β-sheets is driven by a favorable enthalpic contribution, likely associated with differences in packing geometry relative to pleated β-sheets [20]. The hydrogels formed by the blends exhibit a more rigid viscoelastic network than those formed by the corresponding homochiral peptides. In the specific case of MAX1/DMAX1, the increased rigidity of the racemic gel (Table 3) has been attributed to the formation of stiffer fibrils. This behavior can be rationalized in terms of the supramolecular organization dictated by the presence of both peptide enantiomers, which co-assemble into rippled β-sheet structures. This structural organization is characterized by an alternating antiparallel arrangement of MAX1 and DMAX1 peptides within the β-sheets, resulting in an extended network of intermolecular parallel β-strands. In contrast, enantiomerically pure MAX1 assembles into fibrils composed of an extended array of anti-parallel β-strands. The unique organization of the racemic system promotes more efficient packing of the valine side chains, driven by the maximization of van der Waals contacts, which are absent in the pure MAX1 hydrogel [68]. In contrast, the blend of KYFIL-NH_2_ and kyfil-NH_2_ peptides, in which a hydrophobic stretch of amino acids is flanked by a hydrophilic amino acid, forms a hydrogel with lower stiffness than the hydrogel formed by homochiral components (Table 3) [71]. The homochiral peptides assemble into nanoscale fibers that entangle into a three-dimensional gel, whereas the heterochiral blend forms micro-scale plates, which are unable to entangle and therefore yield softer hydrogels. This morphological change can be related to a different molecular packing of the peptides in the blended mixture, as revealed by X-ray analysis. The stereocomplexation in water also induces a conformational transition from random coil (homochiral peptides) to β-sheets (racemic mixture).

When KYFILC-NH_2_ and kyfilc-NH_2_ peptides are conjugated separately to a four-arm PEG, the stereocomplex formed by blending peptide-PEG conjugates of opposite chirality exhibits superior gelation compared to the homochiral PEG conjugates [72]. This phenomenon is attributed to the formation of β-sheets in the stereocomplex, as assessed by IR spectroscopy.

Heterochiral peptides can be generated using different strategies, such as alternating stretches of L- and D-amino acids, alternating L- and D-amino acids or replacing selected L-amino acid with D-amino acids.

The impact of block chirality has been investigated using the peptide KFE8 (Ac-FKFEFKFE-NH_2_): the comparison of homochiral and block heterochiral peptides, such as Ac-FKFEfkfe-NH_2_ or Ac-fkfeFKFE-NH_2_, shows that homochiral peptides assemble into β-sheets and form fibrils while heterochiral peptides adopt a mixture of β-sheets and random conformations, producing helical tapes (Table 3). In block heterochiral peptides, a rotation at the L/D interface occurs, which maintains the hydrogen bond network as well as hydrophobic and electrostatic interactions mediated by the F and K or E residues on the two faces of the β-sheets. The hypothesized molecular model shows that the rotation induced by the change in chirality of the peptide block shifts the phenylalanine side chains from one side of the backbone plane to the opposite one, thereby abolishing the amphipathic character observed in the homochiral peptide, in which alternating residues are all oriented on the same side of the main chain. Therefore, the heterochiral peptide is no longer able to form a bilayer architecture and instead assembles into a monolayer arrangement. Furthermore, the inversion of chirality induces internal strain that offsets the intrinsic twist of the β-sheet, leading to a partial flattened conformation. Rheological studies indicate that homochiral peptide hydrogels possess higher storage moduli compared to those formed by heterochiral peptides (Table 3). All hydrogels show consistent mechanical recovery after multiple cycles of low-high strains [73]. The effect of mixing homochiral and block heterochiral peptides has been recently investigated, using the peptide KFE8 [74]. Mixtures were produced with homochiral peptides, with two blocks showing the same chirality (LL or DD) and heterochiral peptides with the two blocks of different chirality (LD or DL). When the peptides possess the same chirality at the N-terminus (LL/LD and DD/DL) co-assembly occurs. TEM micrographs reveal the presence of helical fibers exhibiting width and helical pitch different from that of pure components. When the peptides possess different chirality at the N-terminus (LL/DL or DD/LD), self-sorting occurs. In this case homochiral fibers and heterochiral tapes appear in the TEM images. The modification at the N-terminus of peptides drastically affects the morphology of the assembled systems. In homochiral KFE8 the lateral aggregation of fibers depends on long-range stacking between N-terminal phenylalanines. These interactions are preserved only when the N-terminal chirality is matched. The corresponding hydrogels have different stiffness; the self-sorting peptide hydrogels are stiffer than the co-assembling peptide hydrogels (Table 3). This is likely due to the different interactions and packing between fibrils and tapes or fibrils.

Another example of block chirality has been reported by Xie and coworkers, in studies aimed at relating the chirality of fibers formed upon peptide self-assembly to their antimicrobial activity. Homochiral peptides, obtained by conjugating the 16-carbon atom chain palmitic acid (C16) to a peptide, to give C16-V_4_R_4_ and C16-v_4_r_4_ and heterochiral C16-v_4_R_4_, were investigated [75]. The peptides form β-sheets further assembling into fibers; the degree of β-sheet twisting is lower in the heterochiral than in the homochiral peptides. The chirality of the valine residues dictates the screw sense of the helical fibers: the L peptide forms left-handed helices, while the D-peptides and the heterochiral peptides form right-handed helical structures. All peptides form hydrogels at pH 11, with the heterochiral peptide producing the least viscoelastic hydrogel. Interestingly, the antibacterial activity differs among enantiomers (see “The biology of chiral peptide hydrogel” paragraph).

**Figure 6:**
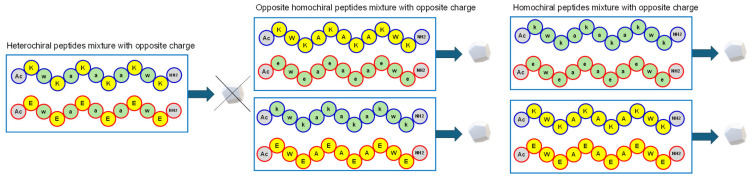
Hydrogel formation by peptide mixture as described by Taraban et al. [76]. L-amino acids are represented as uppercase letters on a yellow background, while D-amino acids are shown as lowercase letters on a green background. Blue outlines indicate positively charged peptides, and red outlines indicate negatively charged peptides. The grey polyhedron represents a hydrogel.

Taraban et al. have described the mechanical properties of hydrogels formed by mixing a positively charged peptide with a negatively charged peptide containing amino acids of the same or opposite chirality (Figure 6 and Table 3) [76]. These peptides are composed of hydrophobic amino acids such as W and A, and hydrophilic amino acids (K or E), Ac-XWXAXAXAXWX-NH_2_ (X = K or E). Heterochiral peptides were obtained using alternating L- and D-amino acids. A mixture of heterochiral peptides does not form a hydrogel. Mixtures of homochiral peptides with opposite charges (one with X = K and the other with X = E) and opposite chirality, as well as opposite charges and the same chirality, form hydrogels. When homochiral peptides with opposite chirality are mixed, gelation occurs faster than for mixtures of peptides with the same chirality. On the contrary, mixtures of peptides with the same chirality produce hydrogels with higher strain yield than mixtures of peptides with opposite chirality. SAXS analyses reveal the formation of elongated asymmetrical assemblies in the gelling mixtures and of oligomers of finite size in the non-gelling pairs. The homochiral pairs form strong hydrogels (Table 3) at a slower rate, which is attributed to fiber morphology. Homochiral pairs form thicker fibers than heterochiral pairs; heterochiral fibers are interconnected by thicker webs as compared to homochiral pairs.

Hydrogels can be obtained through the polymerization of amino acids. The chirality of amino acids critically affects both the polymers’ structure and solubility. For example, polymerization of L-serine results in insoluble polymers; the polypeptide has a strong tendency to precipitate due to its propensity to fold into a β-sheet structure. On the contrary, polymers containing D- and L-serine, obtained after crosslinking acryl-derivatized poly-serine, can be used to obtain porous hydrogels [77]. The alternation of D- and L-amino acids modifies the polymer structure, resulting in a random coil conformation endowed with higher solubility. These hydrogels have been shown to be highly bio-inert and are therefore suitable for implantable biomedical devices, able to mitigate foreign body responses (FBR).

Finally, the effect of chirality on hydrogels composed of peptides and polymers has been explored. Hydrogels obtained upon crosslinking homochiral peptides Fmoc-FFC or Fmoc-ffc by poly(ethylene glycol) dimethyl acrylate (PEGDMA) were characterized [78]. CD profiles show opposite signals, as expected. TEM images reveal fibers of similar dimensions for gels of opposite chirality, and rheological studies reveal that these are soft hydrogels with comparable stiffness (Table 3).

Homochiral and heterochiral peptides sensitive to matrix metalloproteinase (MMP) degradation were conjugated to an eight arm PEG scaffold, with each arm modified with a norbornene. The homochiral peptide is composed of L-amino-acids (Ac-GCRDGPQGIWGQDRCG-NH_2_), the heterochiral peptide (Ac-GCRDGPqGiwGQDRCG-NH_2_) contains D-amino-acids at positions sensitive to protease degradation. Hydrogels were prepared using either each peptide separately or a mixture of the two. All hydrogels obtained after a photochemical reaction show similar physical and mechanical properties (porosity, pore size, and Young’s modulus) (Table 3) but different biological properties [79].

In a different approach, the effect of chirality has been investigated by exploring the properties of block copolymers containing PEG and γ ethyl L- or D-glutamate polymers, named mPEG-b-PELG (with the L amino acid) and mPEG-b-PEDG (with the D-amino acid) (Figure 5C) [80,81]. Conjugation of PEG to the amino acid polymer changes PEG hydrophobicity. The copolymers, synthesized by the ring-opening polymerization of γ-ethyl-(L or D)-glutamate N-carboxyanhydride with a PEG unit, contain homochiral or heterochiral glutamate residues. Surprisingly, the secondary structure of homochiral copolymers, as evaluated by CD experiments, seems to be dependent on the polymerization degree, being a mixture of α-helical and β-sheets structures when the polymerization degree is around 16 [80] and only β-sheets when the polymerization degree is around 13 [81]. The morphology of the copolymers depends on the chirality of the amino acid conjugated and on the relative ratio between L and D amino acids in the enantiomeric mixtures: spherical structures form when the L and D enantiomers are in a 1:1 molar ratio, whereas rod-like structures are observed when one enantiomer predominates. The heterochiral glutamate-based copolymer exhibits the shortest gelation time, the lowest gelation temperature and the highest G’. On the contrary, block copolymers composed of PEG and heterochiral alanine show poorer gelation properties compared to copolymers of PEG and homochiral peptides [82]. The difference in the composition of the amino acid side-chain drastically affects the properties of the hydrogel.

**Table 3:** Sequences and properties of hydrogels formed by long peptides.

Peptide Sequence or Name	Secondary Structure	Minimum Gelation Concentration, Solvent	G′ (Pa)	Morphology	Refs.
Ac-FFFK	β-sheets	3 mM, 298 mM sucrose **	1.5 × 10^2^ *	cylinder nanofibers	[63]
Ac-fffk	β-sheets	3 mM, 298 mM sucrose **	1.5 × 10^2^ *	cylinder nanofibers	
Ac-FFFKTTKS	β-sheets	2.9 mM, 298 mM sucrose **	1 × 10^3^ *	nanofiber	[64]
Ac-fffkttks	β-sheets	2.9 mM, 298 mM sucrose **	2 × 10^2^ *	nanofiber	
(VK)_4_VpPT(KV)_4_-NH_2_	β sheets	4.5 mM, 100 mM BTP, 300 mM NaCl pH 7.4	3 × 10^2^	fibrils	[68,69]
(VK)_4_VpPT(KV)_4_-NH_2_/(vk)_4_vPpt(kv)_4_-NH_2_	rippled β-sheets	4.5 mM, 100 mM BTP+ 300 mM NaCl pH 7.4	7.5 × 10^2^	fibrils	
Ac-(FKFE)_2_-NH_2_	β-sheets	1 mM, water	1.8 × 10^3^	fibrils, helical nanoribbons	[20,70]
Ac-(fkfe)_2_-NH_2_	β-sheets	1 mM, water	1.9 × 10^3^	fibrils, helical nanoribbons	
Ac-(FKFE)_2_-NH_2_/Ac-(fkfe)_2_-NH_2_	rippled β-sheets	1 mM, water	2.7 × 10^3^	tape-like nanofibrils	
KYFIL-NH_2_	β-sheets	2.2 mM, PBS 22 mM **	31 × 10^3^	fibers	[71]
kyfil-NH_2_	β-sheets	2.2 mM, PBS 22 mM **	23 × 10^3^	fibers	
KYFIL-NH_2_/kyfil-NH_2_ (1:1)	β-sheets	4.4 mM, 44 mM PBS **	3 × 10^3^	plates	
KYFILC(PEG)-NH_2_	random coils	7.5% *w/v* in PBS	8	n.d.	[72]
kyfilc(PEG)-NH_2_	random coils	7.5% *w/v* in PBS	12.9	n.d.	
Ac-FKFEfkfe-NH_2_	β-sheets + random coils	10 mM, water *	300	helical tapes	[73]
Ac-fkfeFKFE-NH_2_	β-sheets + random coils	10 mM, water *	200	helical tapes	[73]
Ac-FKFEFKFE-NH_2_/Ac-FKFEfkfe-NH_2_	β-sheets	10 mM, water *	* 10^4^	fibers	[74]
Ac-FKFEFKFE-NH_2_/Ac-fkfeFKFE-NH_2_	β-sheets	10 mM, water *	* 10^5^	fibers + tapes	
C16-V_4_R_4_	β-sheets	7.83 mM, water pH 11	1.3 × 10^3^	helical fibers	[75]
C16-v_4_r_4_	β-sheets	7.83 mM, water pH 11	1.1 × 10^3^	helical fibers	
C16-v_4_R_4_	β-sheets	7.83 mM, water pH 11	15	helical fibers	
Ac-KWKAKAKAKWK-NH_2_/Ac-eweaeaeaewe-NH_2_	n.d.	8 mM, water	5 × 10^3^	fibers	[76]
Ac-kwkakakakwk-NH_2_/Ac-EWEAEAEAEWE-NH_2_	n.d.	8 mM, water	5 × 10^3^	fibers	
Ac-kwkakakakwk-NH_2_/Ac-eweaeaeaewe-NH_2_	n.d.	8 mM, water	9 × 10^4^	fibers	
Ac-KWKAKAKAKWK-NH_2_ Ac-EWEAEAEAEWE-NH_2_	n.d.	8 mM, water	9 × 10^4^	fibers	
PEGDMA- Fmoc FFC crosslinked	n.d.	** 0.8 mM peptide (0.5 wt%), 7.85 mM PBS pH7	200	fibers	[78]
PEGDMA- Fmoc ffc crosslinked	n.d.	** 0.8 mM peptide (0.5 wt%); 7.85 mM PBS pH7	200	fibers	
8armPEG-NB/Ac-GCRDGPQGIWGQDRCG-NH_2_	n.d.	n.d., glycerin	1 × 10^4^	n.d	[79]
8armPEG-NB/Ac-GCRDGPqGiwGQDRCG-NH_2_	n.d.	n.d., glycerin	1 × 10^4^	n.d.	
8arm PEG-NB/Ac-GCRDGPQGIWGQDRCG-NH_2_/PEG-NB/Ac-GCRDGPqGiwGQDRCG-NH_2_	n.d.	n.d., glycerin	1 × 10^4^	n.d.	
mPEG-b-PELG	β-sheets	4.0 wt% water	172	rodlike nanoparticles	[80,81]
mPEG-b-PEDG	β-sheets	5.0 wt% water	269	rodlike nanoparticles	
mPEG-*b*-P(ELG_0.5_-*co*-EDG_0.5_	n.d.	4.0 wt% water	92	spherical nanoparticles	

N.d.: not determined; BTP: bis-tris propane; PBS: phosphate buffer saline; C16 = palmitic acid; NB norbornene unit; *: concentration reported for rheological experiments; **: estimated using molecular weight reported in the literature for the compounds.

## 4. The Biology of Chiral Peptide Hydrogel

**Figure 7:**
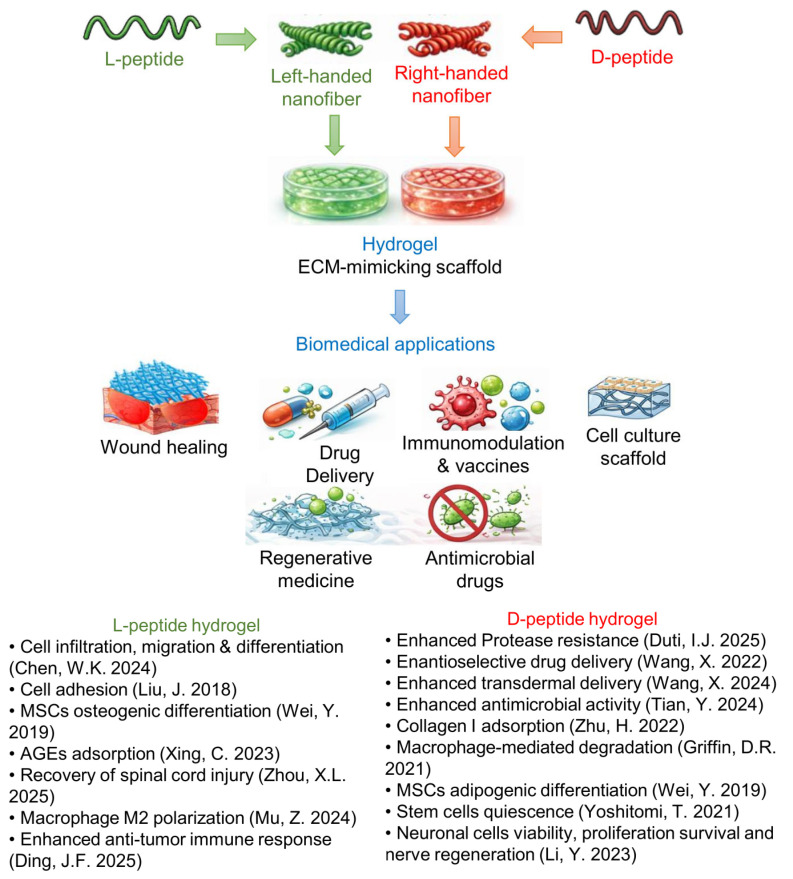
Biological features and applications of chiral peptide hydrogels. Top: L- or D-peptides can self-assemble into supramolecular nanofibers with opposite handedness. These nanofibers further assemble into three-dimensional hydrogel networks that mimic the architecture of the extracellular matrix and can support various biomedical applications. In addition, these materials can be exploited as cell culture scaffolds. Bottom: Examples of biological responses elicited by chiral peptide hydrogels described in this review are shown, with the corresponding references. MSC: mesenchymal stem cells; AGEs: advanced glycation end products [49,50,52,53,63,64,66,72,79,81,86,88,89,90,93].

The biological activity of peptide hydrogels is strongly governed by their molecular and supramolecular chirality. In fact, stereochemical modifications can alter interactions with biological systems. Cell-surface receptors, enzymes, and extracellular matrix components are inherently chiral, and their recognition of peptide-based materials is therefore sensitive to backbone configuration [50]. Understanding how chirality affects biological responses is therefore essential to fully exploit the potential of these materials in biomedical applications. From a medicinal chemistry perspective, one of the primary motivations for incorporating D-amino acids into peptide-based biomaterials is their enhanced resistance to proteolytic degradation. This increased stability can lead to prolonged persistence and functionality in biological environments, providing clear advantages for biomedical applications, such as drug delivery systems. Beyond protease resistance, molecular and supramolecular chirality represents a powerful tool to modulate a broad range of biological properties of peptide hydrogels. D-peptides could not only improve metabolic stability but also expand the spectrum of biological responses that can be elicited, enabling finer control over key cellular processes, including adhesion, migration, proliferation, differentiation, immune modulation, and tissue regeneration. Notably, as reported in the previous paragraphs, even the incorporation of a single D-amino acid within a peptide sequence can profoundly influence peptide self-assembly and supramolecular organization, with direct consequences on biological activity [83]. Furthermore, blending solutions of peptide enantiomers provides an additional level of control to fine-tune hydrogel biophysical properties, such as stiffness, stability, and morphology, which in turn influence cellular behavior and drug release profiles [71]. A growing body of literature has investigated how chiral peptide hydrogels differentially regulate biological processes, including stem cell differentiation, proliferation, cell adhesion and migration, immunomodulation, and tissue repair (Figure 7). These effects arise from the stereoselective recognition of chiral biomaterials by cells or from interaction with components of the extracellular matrix (ECM). Nevertheless, many studies lack direct comparisons between enantiomeric hydrogels, limiting a comprehensive understanding of chirality-driven biological effects. In the following sections, we examine the different biological responses elicited by hydrogels obtained from homo- and heterochiral peptides, as well as by mixtures of L- and D-peptides, highlighting the distinctive properties of each system and their respective advantages for specific biomedical applications.

### 4.1. Protease Stability

Early investigations into the use of D-amino acids in self-assembled peptide hydrogels were motivated by the hypothesis that these materials would display enhanced resistance to enzymatic degradation. In this framework, mirroring peptide stereochemistry has emerged as an effective strategy to extend hydrogel residence time, a key requirement for biomedical applications that demand long-term material persistence [83]. Importantly, the degradability of peptide hydrogels can be finely tuned by adjusting the relative proportions of L- and D-peptides, an approach that is particularly advantageous when these systems are employed as drug delivery platforms [84]. The enhanced proteolytic stability conferred by D-amino acids has been demonstrated in several systems. For instance, PEG-based polypeptide hydrogels incorporating γ-ethyl-D-glutamate, synthesized by the ring-opening polymerization of γ-ethyl-D-glutamate N-carboxyanhydride with a PEG unit, exhibit markedly prolonged enzymatic degradation in vitro, as well as slower degradation in the subcutaneous tissue of rats [80]. Similar benefits have been reported for peptide hydrogels obtained by functionalizing a four-arm PEG scaffold with KYFILC-NH_2_ and kyfilc-NH_2_ peptides [72]. While the conjugation of each enantiomer alone yields materials that remain fully soluble in aqueous media, mixing the L- and D-peptide conjugates in a 1:1 ratio results in robust hydrogel formation with high resistance to proteolytic degradation [72]. In a comparative study of tripeptide hydrogelators with the sequence FxF, Garcia et al. observed that all heterochiral peptides resisted hydrolysis (<20% of degradation over 5 days), with the tripeptides having x = D-Leu and D-Nle showing the best protease resistance [60].

Peptide chirality can also be leveraged to precisely modulate the degradation kinetics of protease-responsive hydrogels, such as MMP-sensitive systems. These materials are particularly relevant for bone regeneration, where controlled degradation is essential to create space for cell infiltration and migration while supporting neovascularization [85]. Chen and coworkers have demonstrated that the degradation behavior of PEG-based peptide hydrogels for bone tissue engineering can be tuned by inverting the chirality of selected amino acids within an MMP-cleavable peptide crosslinker. Hydrogels have been prepared using peptides containing the MMP sensitive sequence GPQGIWGQ or GPqGiwGQ. In particular, the homochiral L-peptide Ac-GCRDGPQGIWGQDRCG-NH_2_, the heterochiral peptide, named D-peptide, Ac-GCRDGPqGiwGQDRCG-NH_2_ or a mixture of L- and D-peptides have been crosslinked to a norbornene-functionalized eight-arm PEG via a thiol–norbornene photoreaction, while an additional peptide (CGRGDSG), containing the RGD motif, has been included to promote cell adhesion. Despite exhibiting comparable mechanical properties, these hydrogels show markedly different degradation rates as a function of peptide chirality [79]. Interestingly, the same MMP-degradable peptides have been employed to prepare degradation-resistant microporous annealed particle (MAP) hydrogels for regenerative wound healing applications [86]. In this case, the inversion of peptide chirality unexpectedly reduces degradation in vitro while significantly accelerating degradation in vivo. This behavior has been attributed to the enhanced recruitment of immune cells, particularly macrophages, within and around the D-peptide crosslinked MAP hydrogel (D-MAP) implants, which trigger a robust immune response and ultimately promote tissue remodeling, skin regeneration, and hair follicle neogenesis [86].

### 4.2. Cytotoxicity, Adhesion, Differentiation, and Tissue Regeneration

Subtle stereochemical modulation, such as the inversion of the chirality of a single amino acid, can critically influence cytocompatibility as well as material architecture. For example, in the context of diphenylalanine-based peptide hydrogels, heterochirality has emerged as an effective design strategy to modulate both supramolecular organization and biological response. The homochiral dipeptide FF, which assembles into heterogeneous microtubes, is cytotoxic, while the hydrogel formed by the heterochiral analogue fF shows a decreased cytotoxic effect as demonstrated in fibroblast and keratinocyte cultures [37]. Moreover, only fF hydrogels form a continuous sheet that detaches from the bottom of culture wells and is preferentially colonized by cells, which proliferate in higher numbers compared to the underlying plastic layer. Similar observations have been reported for the fV dipeptide-based hydrogel [41]. Notably, this latter hydrogelator sequence lacks amyloid character, as confirmed by the absence of thioflavin T fluorescence, and exhibits excellent in vitro biocompatibility, with no cytotoxic effects observed in fibroblast cultures [41]. In addition, although the fV hydrogel partially dissolves and detaches from the plastic surface, cells are found in high numbers within the gel matrix itself. This preferential growth within the hydrogel scaffold, rather than on the underlying plastic further supports the cytocompatibility and biological potential of heterochiral peptide hydrogels. Among the heterochiral tripeptides with sequence FxF, fibroblast viability data revealed no major cytotoxicity, with the greatest cell survival observed with the FlF hydrogel [60]. Similarly, heterochiral tripeptides fFV and Ffv were demonstrated to be biocompatible and to support fibroblast viability and proliferation [58].

Peptide-based hydrogels are widely used as extracellular matrix (ECM) mimetics in biological applications, as they closely recapitulate key features of ECM, including high water content, structural similarity, and permeability that supports nutrient diffusion, metabolic exchange, and cell migration. These properties create a permissive microenvironment for cell adhesion, proliferation, and differentiation, highlighting peptide hydrogels as promising biomaterials for tissue regeneration and wound healing applications [83,87]. Within this framework, peptide chirality can be used to tune cellular behavior and tissue-specific biological responses. Accumulating evidence across multiple cell types indicates that matrices composed of L-peptides generally support adhesion, proliferation, and differentiation, whereas D-peptides often elicit weaker or even inhibitory effects, and therefore can show different applications [52,78,88]. A seminal contribution in this area was provided by Liu and coworkers, who have disentangled the respective roles of molecular and supramolecular chirality using hydrogels based on the LPFEG or DPFEG scaffold. Modifying the number of methylene units (odd or even) between the aromatic ring and the carbonyl switches the handedness of the resulting nanofibers while maintaining the amino acid (L- or D-Phe) configuration constant. This approach enables the independent evaluation of molecular versus supramolecular chirality in regulating cell behavior. Their work reveals that left-handed nanofibers derived from LPFEG induce the most pronounced enhancement of cell adhesion and proliferation. In contrast, right-handed nanofibers formed from LPFEG, as well as left-handed nanofibers assembled from DPFEG, produce only marginal effects on cell adhesion and proliferation. Right-handed nanofibers derived from D-phenylalanine exert instead a negative effect on cell adhesion [52]. This effect could be attributed to stereospecific recognition by cells (e.g., adhesion proteins).

Chiral peptide hydrogels are gaining increasing attention as drug-free wound dressings, for the treatment of chronic and non-healing wounds. Notably, compared to the levorotatory hydrogels, hydrogels derived from DPFEG selectively promote the adsorption of type I collagen [49]. This effect is likely related to their ability to mimic the native right-handed triple-helical architecture of collagen fibers. Enhanced collagen adsorption, in turn, facilitates keratinocyte adhesion, proliferation, and migration via integrin- and YAP-dependent signaling pathways, ultimately accelerating re-epithelialization and wound closure. These properties highlight the therapeutic potential of hydrogels based on right-handed helical superstructures for chronic wound management, including diabetic ulcers [49]. It is noteworthy that chirality-dependent effects can be context-specific. In diabetic wound environments, LPFEG-based hydrogels outperform their DPFEG counterparts in the adsorption of advanced glycation end products (AGEs), which are key contributors to oxidative stress and impaired angiogenesis. To further enhance functionality, the LPFEG-based hydrogel has been modified with a natural cationic antimicrobial hexapeptide (RWRWRW) and cross-linked with hyaluronic acid. The resulting multifunctional chiral scaffold exhibits antibacterial activity, promotes fibroblast proliferation and migration, and stimulates angiogenesis, leading to significantly improved healing of infected diabetic wounds, particularly in terms of vascularization and re-epithelialization [89]. Collectively, these studies underscore the versatility of chiral hydrogels as tunable wound dressings and provide valuable design principles for targeting revascularization in diabetic wound care.

Chirality also plays a pivotal role in stem cell fate determination, where subtle differences in matrix handedness can profoundly influence lineage commitment. Using LPFEG and DPFEG Wei and coworkers have constructed self-assembled chiral hydrogels that form three-dimensional ECM-like microenvironments and exhibit similar biocompatibility toward encapsulated mesenchymal stem cells (MSCs). Despite these similarities, the two enantiomeric matrices selectively direct MSC differentiation toward either osteogenic or adipogenic lineages by activating distinct gene expression programs [50].

Comparable chirality-dependent outcomes have been observed in cross-linked peptide hydrogels, where cross-linking enables the modulation of matrix stiffness to better mimic the mechanical properties of native tissues. For example, hydrogels have been obtained by photo-cross-linking the peptides Fmoc-FFC or Fmoc-ffc, which are cross-linkable derivatives of the Fmoc-FF dipeptide, with PEG dimethacrylate harm [78,90]. Studies on human bone marrow-derived MSCs have revealed that the L-form hydrogel supports cell proliferation, spreading, cell–cell interactions, and both osteogenic and adipogenic differentiation in three-dimensional culture. In contrast, the D-enantiomeric hydrogel markedly suppresses these processes. These observations suggest that Fmoc-ffc hydrogels may be particularly useful when the maintenance of stem cell quiescence or an undifferentiated state is desired, potentially reducing risks associated with uncontrolled proliferation or tumorigenesis in cell-based therapies.

In striking contrast to most non-neural cell types, neuronal cells display a preferential response to matrices composed of D-amino acids. A growing number of studies report enhanced neuronal cell density, viability, survival, and differentiation on DPFEG hydrogels, a phenomenon described as “chirality selection for D-matrices” [88,91,92]. At the molecular level, this effect has been linked to the activation of the JNK and p38/MAPK signaling pathways, triggered by reduced cytoskeletal tension arising from weaker interactions between D-matrices and actin filaments [88]. Importantly, DPFEG-based hydrogels have also demonstrated robust efficacy in peripheral nerve regeneration. Li and coworkers have shown that these materials significantly promote sciatic nerve repair, both with and without stem cell implantation, by enhancing Schwann cell proliferation, functionality, and myelination [88].

A chirality-dependent peptide hydrogel bioactivity has been also observed in retinal progenitor cells (RPCs). In this cell line, only DPFEG hydrogel significantly enhances RPC proliferation via the activation of the Akt and ERK pathways, ultimately leading to neuronal differentiation. Conversely, LPFEG hydrogels preserve RPC stemness. Notably, both enantiomeric matrices exhibit anti-inflammatory effects [91,92]. Altogether, these findings suggest the potential application of P-type nanofiber hydrogels in nerve regeneration and the treatment of neurodegenerative diseases.

Nevertheless, the application of peptide-based hydrogels in nerve regeneration as biomaterials is not limited to those composed of D-amino acids, as it critically depends on material design and the intended mechanism of action. For instance, Zhou and coworkers compared hydrogels formed by peptides Ac-FFFKTTKS and Ac-fffkttks in a spinal cord injury model [64]. The peptide sequence combines the collagen-stimulating pentapeptide KTTKS with three phenylalanine residues that drive self-assembly. The hydrogel based on the L-peptide exhibits superior performance in promoting neuronal regeneration and motor function recovery in vivo.

### 4.3. Enhanced Delivery of Drugs

Chiral peptide hydrogels have been extensively investigated as delivery platforms for chiral drugs, including anticancer agents, by exploiting homochiral attractive interactions to preferentially load a specific drug enantiomer. For example, the hydrogel assembled from DPFEG-GO has been developed for the selective delivery of oxaliplatin through a photothermal and photoresponsive component [53]. In this system, (*1R*, *2R)*-oxaliplatin exhibits preferential loading within the hydrogel network with respect to (1S, 2S)-oxaliplatin. Upon near-infrared irradiation, photothermal heating triggers a supramolecular helical inversion from left-handed to right-handed nanostructures, resulting in rapid drug release and synergistically combining chemotherapy with photothermal therapy.

Chiral supramolecular hydrogels formed from LPFEG and DPFEG gelators have also been explored to enhance transdermal drug delivery [93]. In a recent study, these materials have been employed for the topical administration of sodium aescinate, a bioactive compound with strong therapeutic potential for lymphedema but limited clinical applicability due to low metabolic stability and severe adverse effects associated with intravenous delivery. Owing to the pronounced enantioselectivity of the stratum corneum of the skin, particularly due to the presence of keratin fibers and ceramides, toward left-handed supramolecular architectures, the LPFEG hydrogel, which self-assembles into left-handed helical nanofibers, significantly outperforms its D-counterpart in promoting aescinate skin penetration. This enhanced transdermal transport translates into improved therapeutic efficacy in the treatment of lymphedema [93].

### 4.4. Immunomodulation and Vaccines

Chirality can influence peptide hydrogel immunogenicity. In general, D-peptides are considered less prone to immune recognition due to their reduced compatibility with antigen-processing pathways [94,95]; however, due to their prolonged residence time in vivo, D-peptide hydrogel can exert a higher proinflammatory response, leading to the expression of a higher level of the proinflammatory cytokines such as tumor necrosis factor (TNF-α), interleukin-1β (IL-1β), and interleukin-6 (IL-6) [96]. Besides, supramolecular organization, including β-sheet-rich fibrillar assemblies, may still modulate immune responses in a structure-dependent manner [64,97]. Peptide hydrogels composed of L- and D-amino acids can differentially regulate inflammatory responses and immune system activation, highlighting chirality as a key parameter in immunomodulatory biomaterial design. One of the major immunological processes influenced by chiral hydrogels is macrophage polarization, a central mechanism in immunosurveillance, inflammation, and tissue repair. In particular, the hydrogel obtained from the homochiral Ac-FFFK peptide has been shown to preferentially promote macrophage polarization toward the M2 phenotype, which is associated with anti-inflammatory activity and regenerative functions, compared to the hydrogel obtained from the Ac-fffk peptide [63].

Building on this concept, Yang and coworkers have employed LPFEG and DPFEG gelators to modulate macrophage polarization and investigate its impact on myocardial infarction repair [98]. Their results demonstrate that matrices formed by LPFEG containing left-handed helical nanofibers significantly suppress inflammatory responses and enhance myocardial tissue regeneration, by upregulating M2 macrophage polarization. Mechanistically, this effect is mediated by the enhanced clustering of mechanosensitive integrin β1, leading to the activation of focal adhesion kinase (FAK) and Rho-associated protein kinase, as well as downstream PI3K/Akt1/mTOR signaling pathways [98].

The influence of chirality on immune regulation has also attracted considerable attention in cancer vaccine research. In this context, Ding and coworkers have investigated how peptide hydrogel chirality shapes antitumor immunity and the local immune microenvironment [81]. Using mPEG-b-PELG and mPEG-b-PEDG hydrogels, they have demonstrated that hydrogels formed by mPEG-b-PEDG exhibit higher intrinsic immunogenicity and induce more pronounced immune cell infiltration in vivo. However, this excessive inflammatory response led to the establishment of an immunosuppressive microenvironment, characterized by the upregulation of suppressive markers on antigen-presenting cells and the exhaustion of T cells, ultimately resulting in insufficient antitumor efficacy. In contrast, the more balanced immune activation elicited by mPEG-b-PELG hydrogels generates superior anti-tumor immune responses. This study provides valuable design principles for peptide hydrogel-based vaccine platforms, in which chirality can be strategically tuned to optimize immune activation while avoiding immune exhaustion.

Beyond vaccination, chiral peptide hydrogels have also shown promise in mitigating implantation-induced FBRs. Poly(DL-serine) hydrogels implanted subcutaneously in mice exhibit markedly improved anti-FBR performance compared to conventional PEG-based hydrogels, as evidenced by reduced inflammatory cell infiltration and a lower expression of pro-inflammatory cytokines and genes after implantation [77]. These results further underscore the versatility of chiral peptide hydrogels as biocompatible and immunomodulatory materials with broad potential for biomedical and translational applications.

### 4.5. Antibacterial Activity

Chiral peptide hydrogels have demonstrated significant antibacterial potential. The introduction of D-amino acids into self-assembling antimicrobial peptides (AMPs) can enhance their proteolytic stability, activity, and selectivity, thereby broadening strategies to combat multidrug-resistant pathogens and enabling the development of innovative, infection-resistant scaffolds for tissue engineering. A representative example is the peptide Ac-khhqklvffak (KKd-11), containing the self-assembling klvffak motif together with basic residues to impart antimicrobial activity [65]. Although the two enantiomers of the peptide show the same minimum inhibitory concentration (MIC), KKd-11 has been shown to self-assemble into a hydrogel with superior long-term antimicrobial activity compared to the hydrogel fully composed of L-amino acids, Ac-KHHQKLVFFAK (KK-11), an effect attributed to its enhanced resistance to proteolytic degradation. Notably, additional studies on KKd-11 have demonstrated that it not only inhibits biofilm formation but also disrupts preformed mature biofilms [65]. Similar results have been reported for the peptide (2-Nap)-FIIIKKK (IK1; where Nap is naphthaleneacetic acid) which has been synthesized in the mirror form, composed entirely of D-amino acids (D-IK1) [66]. In addition, since the peptide contains three isoleucine residues, each bearing an additional chiral center in the side chain, a further D-analog has been prepared in which the Ile residues were substituted with D-allo-Ile isomer. The results reveal that both analogs containing D-Ile and D-allo-Ile retain the ability to self-assemble into hydrogels and exhibit significantly enhanced resistance to chemical and proteolytic degradation. Moreover, they show higher antibacterial activity against sensitive bacterial strains, as evidenced by lower MIC values, compared to the L-amino acid-based enantiomer. Notably, the D-peptide analogs also display an improved ability to impair the viability of HepG2 hepatocarcinoma cells. Interestingly, D-IK1 analogs are able to support fibroblast adhesion and proliferation, demonstrating their utility as a 3D scaffold for cell culture applications. Overall, these properties suggest that D-IK1 analogs may represent versatile wound dressings, suitable for use as a cell culture scaffold, for the prevention of multidrug-resistant bacterial infections, the inhibition of tumor recurrence, and the promotion of wound healing [66].

In the context of the antibacterial properties of hydrogels, the interactions between bacteria and chiral peptide-based materials play a significant role. Hydrogels composed of helical nanofibers with different degrees of twist have been successfully obtained through the self-assembly of the homochiral peptides C16-V_4_R_4_ and C16-v_4_r_4_, as well as the heterochiral peptide C16-v_4_R_4_. Self-assembly is driven by the hydrophobic palmitoyl chains (C16) attached to the N-terminus of the β-sheet-forming stretch of four valine residues, while a strong affinity for bacterial cell membranes arises from the cationic four arginine residues at the C-terminus. Compared to the homochiral systems, the heterochiral C_16_-v_4_R_4_ peptide self-assembles into more stable right-handed helical nanofibers with a lower degree of twisting and a larger helical pitch, which exhibits an enhanced membrane-disrupting capability, as confirmed by membrane permeability assays, and consequently higher antimicrobial activity [75]. Lastly, chiral hydrogels derived from LPFEG and DPFEG gelators modified with MTZ and TDZ have shown antibacterial activity against both Gram-positive and Gram-negative bacteria, with enhanced efficacy for TDZ-based systems [54]. Importantly, hydrogels with right-handed nanofibers (D-MTZ and D-TDZ) exhibit higher antibacterial activity than the left-handed counterparts, indicating stereoselective interactions with bacterial cells. The results highlight the amplification of chirality at the supramolecular level as a key factor governing the biological performance of chiral hydrogels.

## 5. Conclusions

The effect of peptide sequence chirality on the physical and biological properties of peptide-based hydrogels arises from a complex interplay of multiple factors, such as peptide length, secondary structure propensity, side-chain interactions (e.g., π-π stacking, van der Waals interactions and hydrogen bonding), and sequence patterning such as amphiphilicity. Within this multifactorial framework, peptide length plays an important role in modulating how chirality influences supramolecular organization. In short peptide sequences, even a single change in chirality can significantly affect intermolecular packing. In particular, heterochiral di- or tripeptides often show reduced solubility in aqueous media and an increased tendency to form ordered fibrillar structures able to yield hydrogels upon interaction with water. These effects can be amplified by local packing constraints and side-chain interactions that dominate at short length scales. In long peptide sequences, the introduction of a few amino acids of different chirality has a less marked effect, as the overall folding and assembly are governed by more distributed interactions along the sequence. However, when the percentage of amino acids of opposite chirality is significant (around 50%), long peptides tend to assume a more disordered structure than their homochiral counterparts. This often results in amorphous supramolecular structures, with a lower tendency to form a hydrogel. The physical properties of hydrogels, such as stiffness and porosity, are instead related to the density of the 3D network formed by supramolecular structures, such as fibrillar or tubular architectures. The application of peptide-based hydrogels as biomaterials has been widely explored, particularly for hydrogels composed of long peptides. Conversely, hydrogels formed by short, uncapped peptides often rely on relatively weak non-covalent interactions and tend to be unstable in media rich in salts or nutrients, such as cell culture media. Although efforts toward the improvement of the mechanical properties of hydrogels composed of short peptides have been reported, the effect of chirality on the stability of these systems remains poorly investigated.

## Data Availability

No new data were created or analyzed in this study. Data sharing is not applicable to this article.
